# Medical education with large language models in ophthalmology: custom instructions and enhanced retrieval capabilities

**DOI:** 10.1136/bjo-2023-325046

**Published:** 2024-05-07

**Authors:** Mertcan Sevgi, Fares Antaki, Pearse A Keane

**Affiliations:** 1 Institute of Ophthalmology, University College London, London, UK; 2 Moorfields Eye Hospital NHS Foundation Trust, London, UK; 3 The CHUM School of Artificial Intelligence in Healthcare, Montreal, Quebec, Canada; 4 NIHR Moorfields Biomedical Research Centre, London, Greater London, UK

**Keywords:** Medical Education

## Abstract

Foundation models are the next generation of artificial intelligence that has the potential to provide novel use cases for healthcare. Large language models (LLMs), a type of foundation model, are capable of language comprehension and the ability to generate human-like text. Researchers and developers have been tuning LLMs to optimise their performance in specific tasks, such as medical challenge problems. Until recently, tuning required technical programming expertise, but the release of custom generative pre-trained transformers (GPTs) by OpenAI has allowed users to tune their own GPTs with natural language. This has the potential to democratise access to high-quality bespoke LLMs globally. In this review, we provide an overview of LLMs, how they are tuned and how custom GPTs work. We provide three use cases of custom GPTs in ophthalmology to demonstrate the versatility and effectiveness of these tools. First, we present ‘EyeTeacher’, an educational aid that generates questions from clinical guidelines to facilitate learning. Second, we built ‘EyeAssistant’, a clinical support tool that is tuned with clinical guidelines to respond to various physician queries. Lastly, we design ‘The GPT for GA’, which offers clinicians a comprehensive summary of emerging management strategies for geographic atrophy by analysing peer-reviewed documents. The review underscores the significance of custom instructions and information retrieval in tuning GPTs for specific tasks in ophthalmology. We also discuss the evaluation of LLM responses and address critical aspects such as privacy and accountability in their clinical application. Finally, we discuss their potential in ophthalmic education and clinical practice.

## Introduction

 Foundation models are a new paradigm for building artificial intelligence (AI) systems. They have gained considerable traction due to improvements in computing power, the development of transformer model architecture and the availability of large datasets.^1^) One area that has seen significant utility is natural language processing (NLP), with the advent of large language models (LLMs).^2^) LLMs are foundation models that are trained on large corpora of text, which gives them the capability of language comprehension and the ability to generate human-like text.^1 3^) A notable example is generative pretrained transformers (GPTs).^3^)

There has been growing interest in evaluating the role of LLMs in healthcare.^4^) Despite not being specifically trained in medical knowledge, LLMs have shown generalisation capacities in many domains, including medical challenge problems.^5^) As LLMs are updated, their performance in answering medical examination questions is improving, including in the field of ophthalmology.^46^,^11^) However, since those generic models may not be trained on vetted medical information, their deployment for patient care will be a challenge.^12^) To address this, generic LLMs can be tuned with domain-specific information. For example, models such as BiomedBERT and BioGPT have been trained on content from peer-reviewed literature and Med-PaLM on clinical question databases.^4 13 14^) Models can also undergo task-specific tuning, such as instruction tuning, as is the case with Med-PaLM 2. Those approaches have shown considerable gains in biomedical NLP tasks, including answering medical examination questions. ^7 15^) Prompting strategies (different ways of querying the model) can also be used to augment models. An example of this is Medprompt. It uses innovative prompting strategies with GPT-4 to surpass Med-PaLM 2, which uses domain-specific and task-specific tuning in answering medical examination questions.^5^)

As valuable as these solutions are, there is still the issue of not capturing the most up-to-date information as the training and tuning are performed with a snapshot of data up to a certain point in time.^16^) Due to the extensive volume of data and the time-consuming nature of the training process, there is an inherent delay in updating the knowledge base of LLMs. This is particularly significant in the field of medicine, where up-to-date knowledge and evidence-based practice are fundamental to quality healthcare. Recently, some LLMs were augmented with real-time internet browsing abilities, allowing them to search the internet for up-to-date content to formulate responses.^17 18^) Up-to-date medical information is a pertinent feature of evidence-based medicine,^19^) and thus having real-time information is a crucial feature in LLMs for use in a clinical setting.

In November 2023, OpenAI introduced a feature allowing users to customise their own GPTs using natural language.^20^) During this interaction, GPT developers can provide custom instructions to the GPT to determine its function, interaction with users, method of answering questions, tone and how it retrieves information. GPT can be instructed to use external tools such as internet searches and/or documents uploaded by the developer. In effect, this leverages custom GPTs for their natural language abilities and information retrieval capacity from predetermined sources. This degree of customisation can help overcome issues with LLMs providing false information and reduce inaccuracies if there is more reliance on using trusted, predetermined sources.^21^)

In this review, we provide three use cases of custom GPTs in the field of ophthalmology and how clinicians may interact with these tools. First, ‘EyeTeacher’ is a teaching tool that creates questions from selected clinical guidelines. Second, a clinical assistant, ‘EyeAssistant, answers clinical queries, tuned to clinical guidelines. Third, ‘The GPT for GA’ can provide clinicians with an overview of the current management of geographic atrophy (GA) by retrieving information from peer-reviewed documents uploaded to the GPT. Through these illustrative examples, we show how careful custom instructions and information retrieval are able to tune ChatGPT to specific tasks. We also review how LLMs can be evaluated and explore the privacy and accountability of using these tools in clinical practice.

## Customising LLMs

LLMs are trained on large corpora of text, amassing over a trillion words.^3 22^) Many models have emerged and are accessible through their respective interfaces, such as Gemini (by Google), GPT-4 (by OpenAI), Claude 2 (by Anthropic) and LLaMA (open-access by Meta AI).^23^) These models can be fine-tuned with domain-specific information; however, this requires technical programming expertise. An example of a fine-tuned model is Neuro-GPTx, which is a content-enriched chatbot for the management of vestibular schwannoma trained on over 4000 peer-reviewed articles. ^24^) Another example is Almanac, a medicine-based model that acquires domain-specific knowledge from textbooks and preselected web documents, as well as browsing predetermined web domains.^21^) Also embedded into the model architecture is a calculator, which can overcome the limitations of models being unable to count. These features can improve accuracy and reliability in answering clinical scenario questions.^21^)

Customising LLMs encompasses a variety of approaches, including data-driven strategies and those focused on interaction-level enhancements.^25^) These methods can be effectively combined to optimise model performance and adaptability. At the data level, a pretrained LLM can be adjusted using a dataset of labelled examples through supervised tuning or fine-tuning.^26^) This method requires substantial amounts of high-quality input–output pairs to create a bespoke model tailored for specific responses in a desired domain. Although typically requiring technical expertise, more accessible tuning methods have emerged, such as within the Google ecosystem.^27^) For example, Vertex AI enables users to upload JSON Lines files within an automated machine learning framework to tune an LLM.

At the interaction level, prompt engineering and retrieval augmented generation (RAG) can provide further customisation through interaction with user inputs and external sources of information, respectively. Prompt engineering (and custom instructions) guide model behaviours through natural language instructions, making them more adaptable to specific user needs.^28^) This is enabled by the ability of LLMs to temporarily learn from instructions without changing their internal parameters.^3 29^) RAG, on the other hand, enables LLMs to augment their responses by grounding their knowledge on external sources.^30^) LLMs would retrieve knowledge from a set of fixed sources, such as documents, similar to what is seen in OpenAI’s Custom GPTs. Beyond the OpenAI ecosystem, other user-friendly tools have surfaced, including Cohere AI and Google’s NotebookLM.^31 32^)

In this review, we primarily focus on how GPT models can be customised using natural language for custom instructions and employing user-friendly interfaces for RAG through document uploads. This approach aligns particularly well with healthcare applications, where maintaining factual correctness, professional tone and adherence to clinical guidelines is paramount.

## Custom GPTs

**Figure 1:**
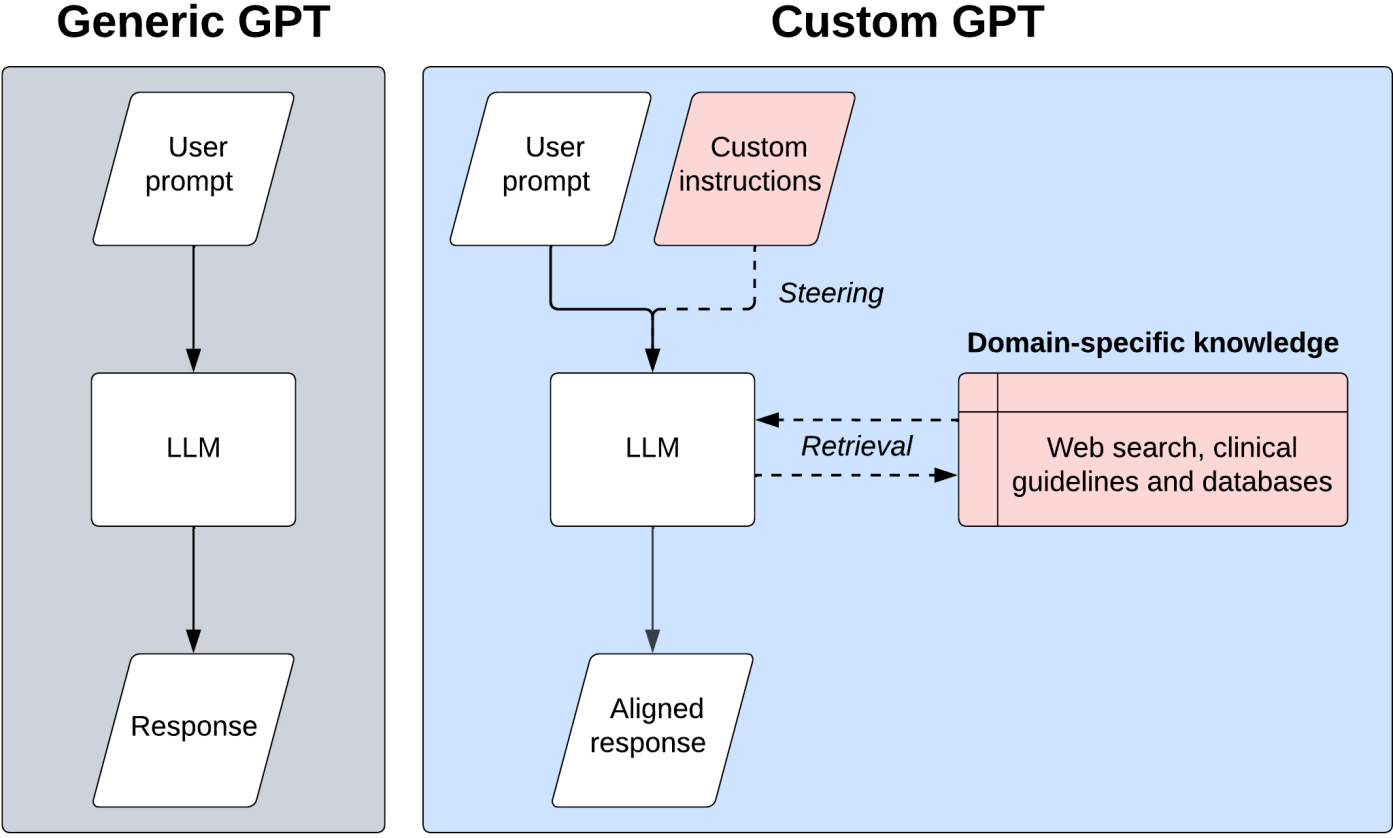
Simplified comparison between generic and custom GPT architectures. Generic GPTs operate linearly: a user prompt is processed by an LLM to generate a response. Custom GPTs integrate custom instructions and domain-specific knowledge into the process. Here, a user prompt is combined with custom instructions (provided by the developer) to steer the LLM, which can also retrieve external knowledge (eg, web searches, clinical guidelines and databases) to produce an aligned response. GPT, generative pretrained transformer; LLM, large language model.

**Figure 2:**
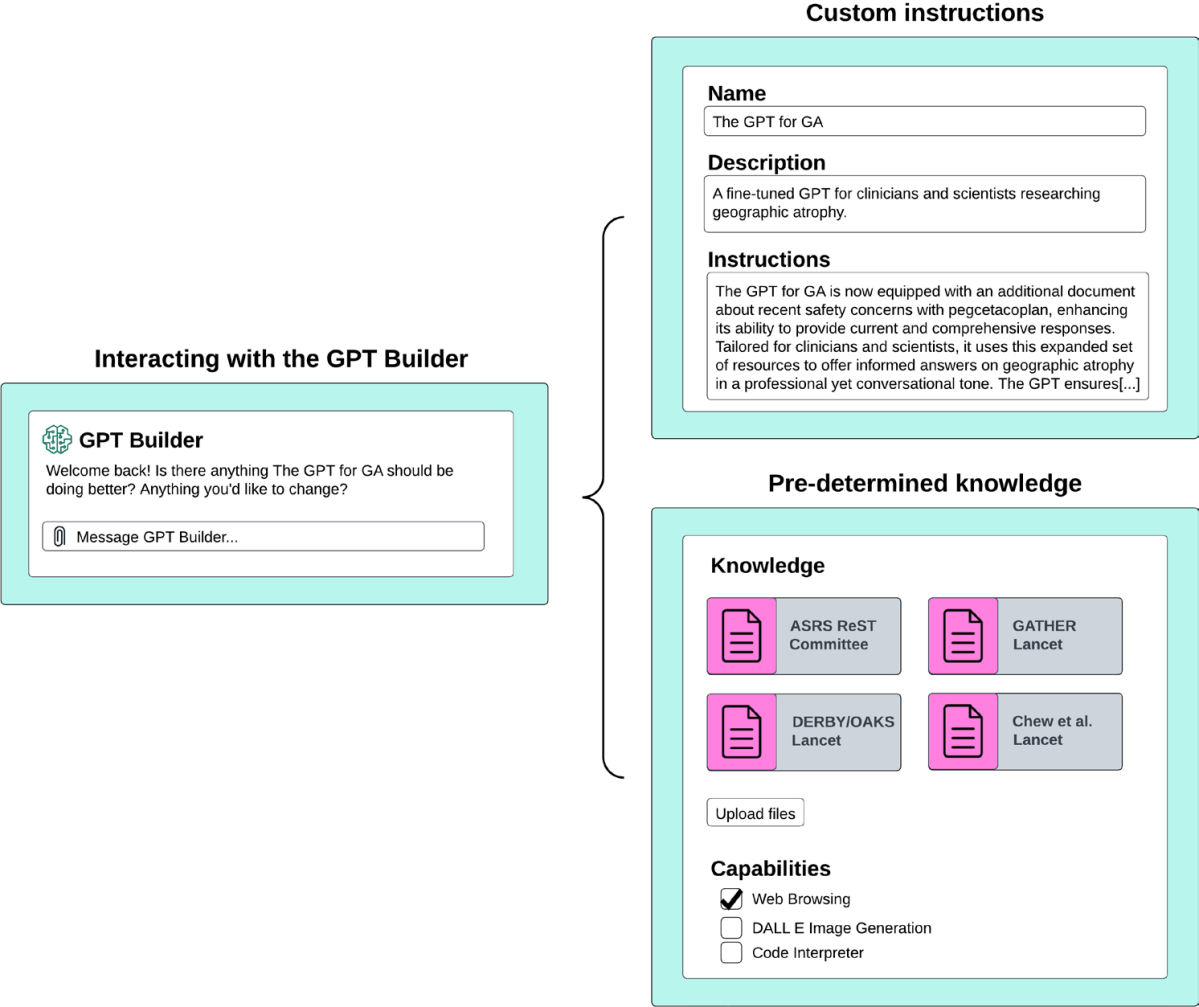
Custom GPTs are built in natural language using a builder chatbot. The GPT builder enables users to input custom instructions using natural language and upload specific knowledge datasets for retrieval. These modifications are implemented in the GPT‘s backend. Additionally, the builder offers the option to enhance or limit functionalities, such as web browsing, image creation and code interpretation and generation, although the latter may be less relevant for our specific use cases. GPT, generative pretrained transformer.

Tuning custom GPTs involves custom instructions with natural language and content retrieval through RAG. We provide an overview of how custom GPTs work in figure 1. ‘GPT Builder’ is the backend for custom GPTs, allowing users to tune the model using natural language, as shown in figure 2. Currently, up to 10 documents may be provided for RAG, with a token limit of 2 million per document. ^33^) Tokens are a unit of analysis employed by LLMs to process text. One token is roughly equivalent to four characters, which corresponds to 0.75 words.^34^) A limit of 2 million tokens per document translates to approximately 1.5 million words, which is more than sufficient for the majority of clinical documents, guidelines and textbooks.

Custom instructions allow for a more steerable model to align with specific user intentions. For use in healthcare, one may want the model to uphold factual correctness, professional tone, privacy and confidentiality, limitation acknowledgement and risk communication, to name some examples. Regarding content retrieval, LLMs are prone to fabricate information, which is commonly termed ‘hallucinations’. ^35 36^) This is a concern for clinical use and will be a limiting factor in LLM deployment. RAG may improve LLM outputs and decrease hallucinations by grounding the model on external sources of knowledge to supplement the LLM’s internal representation of ophthalmology.^37^) For our custom GPTs, we align with the values of evidence-based medicine, providing peer-reviewed literature and clinical guidelines as domain-specific knowledge.^38^) Clinical guidelines are particularly valuable as they represent the consensus of an expert group from a particular organisation. ^39 40^). By tailoring the custom GPT to clinical guidelines, we can provide region-specific domain knowledge that is applicable to the healthcare setting we work in.

## Use cases

### Education: active learning with generated questions

**Figure 3:**
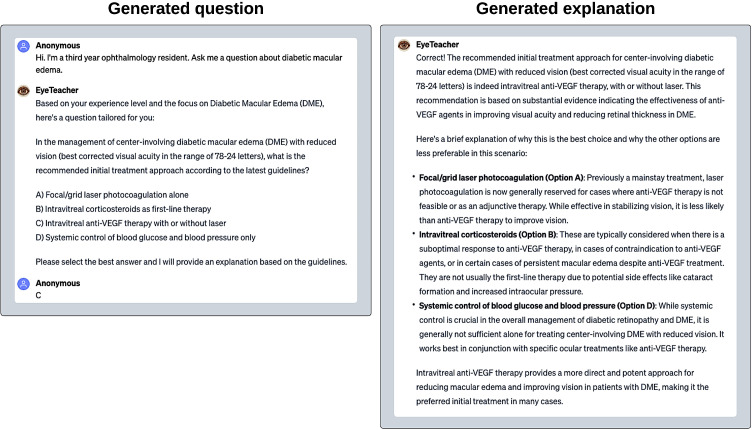
EyeTeacher is a custom GPT built for education. In this enhanced learning scenario, EyeTeacher presents a multiple-choice question about diabetic macular oedema. When a student selects an answer, EyeTeacher elaborates on why the answer is correct and explains why each distractor is incorrect. This approach is crucial because it does not merely validate the student’s choice but educates them about the nuances of each option. This method promotes active learning, as students do not just memorise the right answer; they understand the rationale behind each option. The explanations are free of hallucinations and factually correct. GPT, generative pretrained transformer.

‘EyeTeacher’ demonstrates how users can enhance their understanding of clinical guidelines by answering questions generated by the custom GPT. As an active learning tool, this method of learning can promote more effective knowledge acquisition and retention.^41^) As exemplars, we provide two clinical guidelines on the management of diabetic retinopathy (DR) and angle closure glaucoma (ACG) from the Royal College of Ophthalmologists.^42 43^) We chose these topics as they are common conditions relevant to general ophthalmologists. Both guidelines are within the token limit of the custom GPT (DR: 147 pages, approximately 66 000 tokens. ACG: 44 pages, approximately 19 000 tokens). We showcase an example in figure 3.

We instructed the custom GPT to be an interactive, active learning tool, asking multiple-choice questions with a single best answer. Questions would be generated from the uploaded documents. We also instructed the GPT to ask for the level of experience of the user and to tailor the difficulty of the questions to the user’s level of experience. In keeping with the format of medical question books, we instructed the GPT to provide explanations for the answer. For content retrieval, the GPT is instructed to create the questions from the documents provided. The ability to engage with EyeTeacher is akin to conversing with a personal tutor. It provides flexibility for users to direct their own learning. They can ask for easier or harder questions. They are able to select topics and ask for clarity on the topic if required. The example we have provided is from a clinical guideline; however, one could also use this method to study for exams by uploading notes and asking questions about a specific topic.

### Clinical assistant: interacting with clinical guidelines

**Figure 4:**
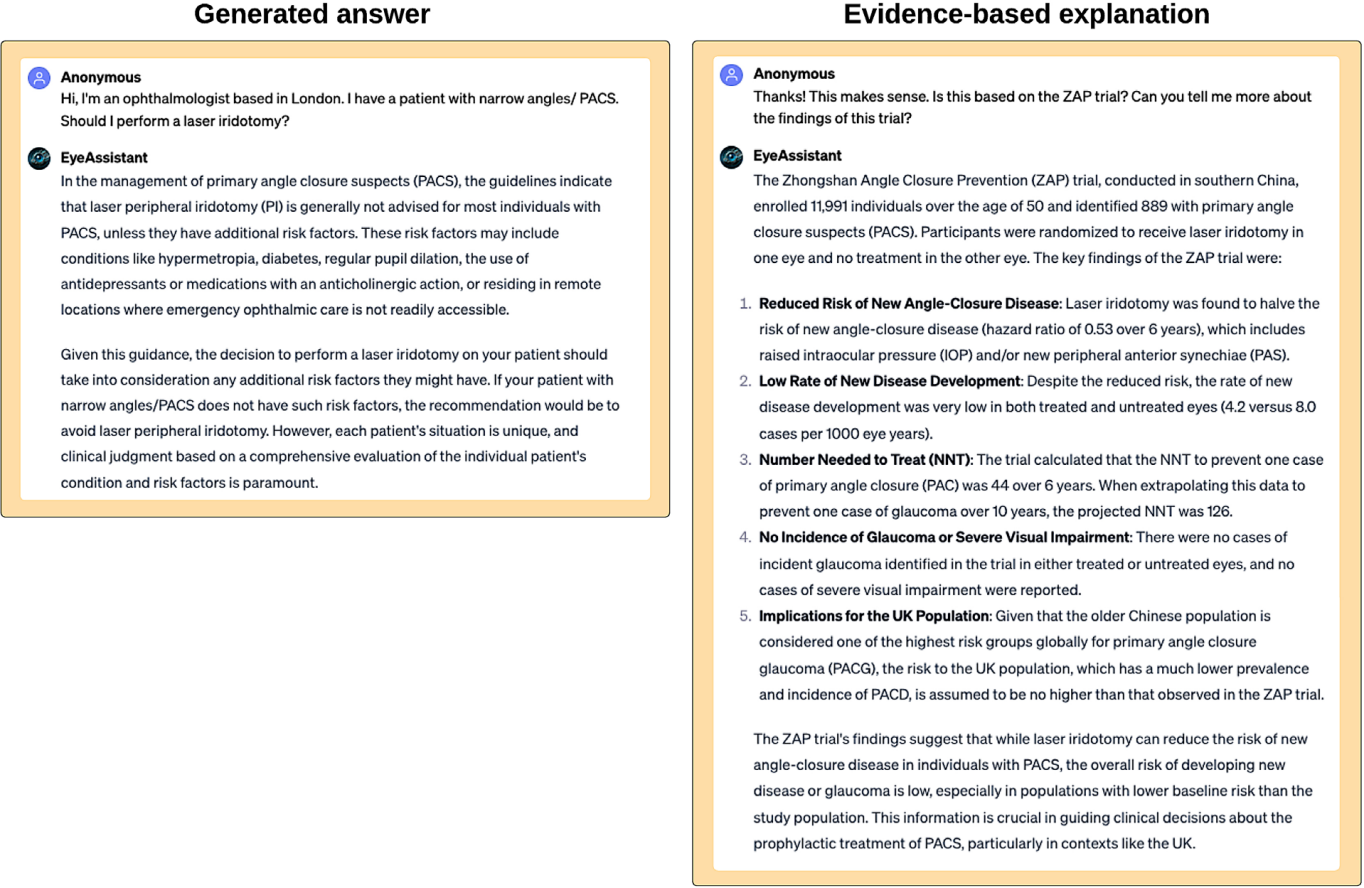
EyeAssistant is a custom GPT built for clinical support. In this clinical scenario, EyeAssistant presents an evidence-based response regarding the management of narrow angles or primary angle closure suspects. From the guideline, it identifies risk factors for angle closure that may warrant prophylactic iridotomy. When prompted to justify the answer and describe the popular Zhongshan Angle Closure Prevention trial, it summarises the findings adequately and contextualises the findings for the London-based ophthalmologist. The explanations are free of hallucinations and factually correct. GPT, generative pretrained transformer

‘EyeAssistant’ is provided with the same clinical guidelines as EyeTeacher, but has separate custom instructions, leading to a different use case and user experience. The purpose of this use case is to act as a clinical assistant. The user is able to converse with the assistant, who has domain-specific knowledge from clinical guidelines. Clinicians would typically query guidelines by reading through them; however, EyeAssistant provides an interactive experience. Users can ask for specific sections in the guidelines as well as provide clinical information such as examination findings or demographic information. EyeAssistant will browse and retrieve the relevant content. To improve reliability, we can include custom instructions in the GPT to ask for further clarification if the information provided is not sufficient to generate an answer. We provide an example in figure 4.

### Summarising peer-reviewed research with custom GPTs

Custom GPTs have emerged as powerful tools for summarising and extracting knowledge from peer-reviewed articles. These custom models offer several advantages over traditional methods of literature review, including enhanced accessibility, efficient knowledge discovery and personalised learning. One of the primary benefits of using custom GPTs for summarising peer-reviewed research is their ability to transform complex scientific literature into concise and understandable language. This makes the information more readily accessible to a wider audience, including researchers, clinicians and even patients. By distilling the key findings, conclusions and implications of research articles, custom GPTs can bridge the gap between scientific experts and those seeking to understand the latest advancements in their field.

In our use case example, ‘The GPT for GA’ summarises peer-reviewed articles on the latest treatment for GA. Custom instructions include tailoring responses to clinicians and scientists and offering informed answers on GA in a professional yet conversational tone. It will focus on providing accurate and relevant information while avoiding speculation and not offering patient-specific medical advice. For content retrieval, The GPT for GA exclusively retrieves information from the provided documents, including the new pivotal GA trials.^44 45^) At the time of writing, there is a limit of 10 document uploads; however, as we expect further advancements in the capabilities of custom GPTs, we will likely see an increase in this capacity.

**Figure 5:**
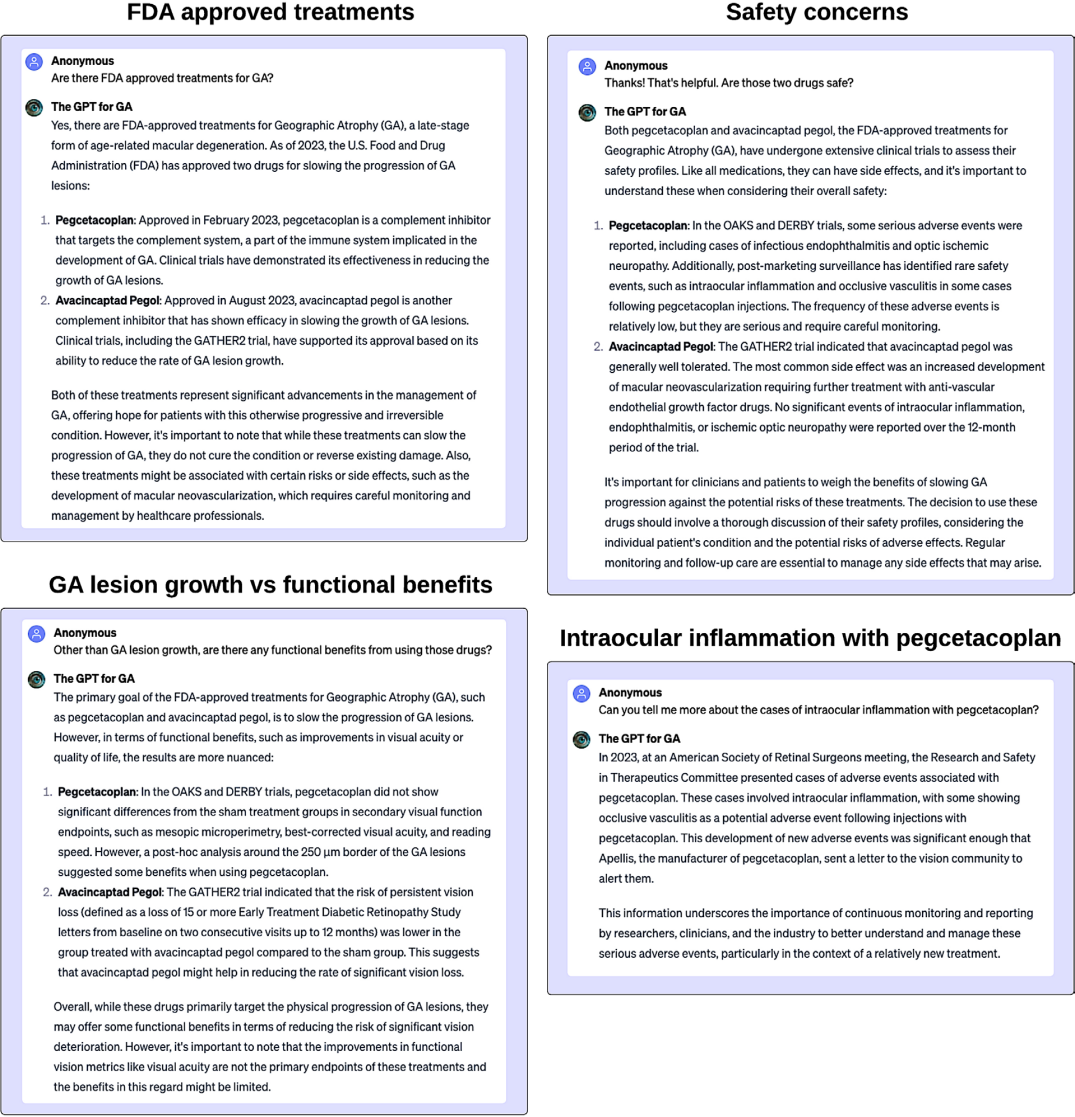
The GPT for GA is a custom GPT built to provide balanced information on the treatments for geographic atrophy. When queried about FDA-approved treatments for GA, The GPT for GA accurately identifies pegcetacoplan (Syfovre) and avacincaptad pegol (Izervay). It appropriately references the pertinent pivotal trials that led to their approval. When prompted to discuss the functional benefits of these treatments, it judiciously cites relevant sources while acknowledging that the associated functional benefits remain limited. Addressing safety concerns, it correctly highlights the elevated risk of macular neovascularisation associated with both drugs and the potential for intraocular inflammation with pegcetacoplan. When asked about the latter, it also provides up-to-date information and cites authoritative sources, such as the American Society of Retina Specialists (ASRS) ReST committee. Of note, it mistakenly refers to the ASRS as the American Society of Retinal Surgeons. FDA, food and drug administration; GA, geographic atrophy; GPT, generative pretrained transformer.

The treatment for GA is an interesting example, as there are many clinical trials underway to find a treatment.^46^), Recently, new drugs have been approved to treat GA. ^44 45^) However, there is concern about adverse events from the newly approved medications.^47 48^) Thus, ‘The GPT for GA’ is able to summarise the most recent literature, providing a balanced overview to readers. We provide an example in figure 5.

## Evaluating responses

The use of LLMs for healthcare requires models that can avoid hallucinations and acknowledge their limitations.^49^) Companies developing LLMs are aware of this and developing models with increased ‘honesty’. For example, Anthropic released Claude 2.1. The company reported that the number of hallucinations was halved compared with their previous version.^50^) In addition to providing fewer incorrect answers, Claude 2.1 has an increased number of responses where it declines to answer due to a lack of knowledge. Another example is Almanac. This model is evaluated on factuality, which can be improved with the use of real-time internet search retrieval and calculators. The completeness and safety of responses are improved with custom instructions.^21^)

Currently, there is no official consensus for evaluating models and responses; however, we can gather insight from studies that evaluate LLMs on answering medical exam questions as well as summarising medical literature.^15 51^) Alongside assessing factual correctness, responses are assessed across multiple evaluation metrics such as comprehension, coherence, knowledge recall, reasoning, potential for harm and relevance, among others.^4^) LLM responses were compared against human responses using these evaluation metrics, which also found that reviewers preferred LLM responses compared with human experts.^15 24 51^) LLMs can be adjusted to perform in varying creative settings. Evaluating ChatGPT-4 explanations of answers for ophthalmology exam questions revealed that more creative settings are preferred.^11^)

## Maintaining safety and accountability

The increasing adoption of LLMs in healthcare raises a critical question: who should be held accountable for any adverse outcomes arising from their use? Within the existing legal framework, clinicians bear the ultimate responsibility for patient outcomes. ^52^) Clinicians should exercise the same degree of caution with LLMs as they do with other medical tools until their efficacy and safety have been rigorously validated. If clinicians wish to use LLMs to aid in their work, tuning with custom GPTs could improve relevance. Uploading their own documents and setting strict instructions are methods users can apply to access more reliable and relevant information. The privacy of data is paramount in healthcare. If clinicians are entering patient information when seeking guidance from LLMs, we need to consider how this information can be kept private. Some LLM providers are offering secure enterprise services so that conversations with the LLMs are not used to train the model and are encrypted.^53 54^) Including privacy controlling measures, there needs to be regulatory oversight for using LLMs in healthcare.^55^) This would require a framework that can evaluate LLMs for their NLP, translational value and governance model to ensure fairness, transparency, trustworthiness, accountability and privacy. ^56 57^)

## Limitations of custom GPTs

The innovative use of custom GPTs in medical education, workflow improvement and clinical assistance has demonstrated considerable potential. However, it is crucial to acknowledge the inherent limitations of these tools to ensure their responsible and effective use.

While a tool like EyeTeacher can facilitate metacognitive learning through generated questions, its effectiveness hinges on the accuracy of the information generated. There is a risk of reinforcing incorrect knowledge if the model generates erroneous content. There is also the risk that EyeTeacher may not probe areas of weakness unless the student explicitly communicates them. In parallel, EyeAssistant, designed to interact with clinical guidelines, may lead to automation bias, where users overrely on AI-generated responses.^58^) Incorrect interpretations or incomplete retrieval of information from the guidelines could lead to misguided clinical decisions. Additionally, the models’ abilities to accurately retrieve information might vary depending on the structure of the documents and the content placement.^59^,^61^)

For The GPT for GA, the current limitation of the number of document uploads restricts the breadth of information that can be summarised. In addition, the static form of content retrieval might miss out on the latest research developments. Updating this would require manual user uploads of the latest research. This underscores the benefit of leveraging real-time internet browsing abilities for LLMs. Indeed, the recent approval of new drugs for GA and concerns about their adverse events highlight the need for timely and comprehensive data integration. Finally, while custom instructions and prompt engineering offer flexibility, they are subjective and highly dependent on the user’s ability to craft effective prompts. This evolving field requires a nuanced understanding of how LLMs interpret and respond to various prompts. Inaccuracies or spurious features in prompts can lead to misleading or irrelevant responses.^62^) Beyond these specific use cases, general limitations of LLMs include their dependence on the training data and its potential biases, and their inability to verify the factual accuracy of generated content. This necessitates careful consideration and validation in contexts where accuracy and reliability are critical, such as in medical and educational applications.

## Conclusion

The rapid evolution of foundation models, particularly in the realm of medical applications, is an exciting development to witness. As researchers and developers continue to refine these models with specialised tuning techniques, we are moving closer to achieving more suitable models for healthcare applications. The future iterations of foundation models and their potential to generalise in solving medical challenges present an intriguing prospect that could pave the way towards artificial general intelligence. The advent of custom GPTs represents a significant step in democratising these powerful tools, thus enabling a wider array of applications. However, further research is essential, particularly in translating these advancements into practical medical settings. We should next focus on medical challenges that mirror actual clinical practice to validate the real-world utility of these models. With the advent of custom GPTs, a multitude of users now have the opportunity to test and adapt these models. The path forward calls for robust research and collaborative efforts to fully harness the potential of custom GPTs in real-world practice. We are at the threshold of a new era in medical technology, an era where AI not only complements but significantly enhances our healthcare capabilities. This is a thrilling prospect, beckoning for further exploration and innovation in the field.

## Data Availability

Data are available upon reasonable request.

## References

[1] Bommasani R, Hudson DA, Adeli E On the opportunities and risks of foundation models. http://arxiv.org/abs/2108.07258.

[2] Nath S, Marie A, Ellershaw S (2022). New meaning for NLP: the trials and tribulations of natural language processing with GPT-3 in Ophthalmology. Br J Ophthalmol.

[3] Brown TB, Mann B, Ryder N (2020). Language models are few-shot learners. https://proceedings.neurips.cc/paper/2020/file/1457c0d6bfcb4967418bfb8ac142f64a-Paper.pdf.

[4] Singhal K, Azizi S, Tu T (2023). Large language models Encode clinical knowledge. Nature.

[5] Nori H, Lee YT, Zhang S (2023). Can generalist foundation models outcompete special-purpose tuning? case study in medicine. http://arxiv.org/abs/2311.16452.

[6] Antaki F, Touma S, Milad D (2023). Evaluating the performance of Chatgpt in Ophthalmology: an analysis of its successes and shortcomings. Ophthalmol Sci.

[7] Taylor R, Kardas M, Cucurull G (2022). Galactica: A large language model for science. http://arxiv.org/abs/2211.09085.

[8] Mihalache A, Huang RS, Popovic MM (2023). Performance of an upgraded artificial intelligence Chatbot for ophthalmic knowledge assessment. JAMA Ophthalmol.

[9] Teebagy S, Colwell L, Wood E (2023). Improved performance of Chatgpt-4 on the OKAP examination: A comparative study with Chatgpt-3.5. *Journal of Academic Ophthalmology*.

[10] Raimondi R, Tzoumas N, Salisbury T (2023). North East Trainee research in Ophthalmology network (Netrion). comparative analysis of large language models in the Royal college of Ophthalmologists fellowship exams. Eye.

[11] Antaki F, Milad D, Chia MA (2024). Capabilities of GPT-4 in Ophthalmology: an analysis of model entropy and progress towards human-level medical question answering. Br J Ophthalmol.

[12] Clusmann J, Kolbinger FR, Muti HS (2023). The future landscape of large language models in medicine. Commun Med (Lond).

[13] Gu Y, Tinn R, Cheng H (2020). Domain-specific language model Pretraining for BIOMEDICAL natural language processing. http://arxiv.org/abs/2007.15779.

[14] Luo R, Sun L, Xia Y (2022). Biogpt: Generative pre-trained transformer for BIOMEDICAL text generation and mining. Briefings in Bioinformatics.

[15] Singhal K, Tu T, Gottweis J (2023). Towards expert-level medical question answering with large language models. http://arxiv.org/abs/2305.09617.

[16] OpenAI (2023). GPT-4 technical report. http://arxiv.org/abs/2303.08774.

[17] OpenAI (2023). Chatgpt Plugins. https://openai.com/blog/chatgpt-plugins#browsing.

[18] Google AI (2023). Bard updates: the latest bard news and AI features - Google bard. https://bard.google.com/updates.

[19] Sackett DL, Rosenberg WM, Gray JA (1996). Evidence based medicine: what it is and what it isn’t. BMJ.

[20] OpenAI (2023). Introducing Gpts. https://openai.com/blog/introducing-gpts.

[21] Zakka C, Shad R, Chaurasia A (2024). Almanac — retrieval-augmented language models for clinical medicine. *NEJM AI*.

[22] Touvron H, Lavril T, Izacard G (2023). Llama: open and efficient foundation language models. https://github.com/facebookresearch/llama.

[23] Yang J, Jin H, Tang R Harnessing the power of Llms in practice: A survey on Chatgpt and beyond. ACM Trans Knowl Discov Data.

[24] Guo E, Gupta M, Sinha S (2024). neuroGPT-X: toward a clinic-ready large language model. J Neurosurg.

[25] Ovadia O, Brief M, Mishaeli M (2023). Fine-tuning or retrieval? comparing knowledge injection in Llms. http://arxiv.org/abs/2312.05934.

[26] Naveed H, Khan AU, Qiu S (2023). A comprehensive overview of large language models. http://arxiv.org/abs/2307.06435.

[27] Google Cloud (2024). Tune language foundation models. https://cloud.google.com/vertex-ai/docs/generative-ai/models/tune-models.

[28] White J, Fu Q, Hays S (2023). A prompt pattern catalog to enhance prompt engineering with Chatgpt. http://arxiv.org/abs/2302.11382.

[29] Wei J, Tay Y, Bommasani R (2022). Emergent abilities of large language models. http://arxiv.org/abs/2206.07682.

[30] Martineau K, IBM Research Blog (2023). What is retrieval-augmented generation?. https://research.ibm.com/blog/retrieval-augmented-generation-RAG.

[31] (2024). Cohere. https://cohere.com/.

[32] Bowen CD, Summersill AR, Google AN (2023). Exploring black undergraduate students' communication and biology education experiences about COVID-19 and COVID-19 vaccines during the pandemic. CBE Life Sci Educ.

[33] OpenAI (2023). File Uploads with Gpts and advanced data analysis in Chatgpt. https://help.openai.com/en/articles/8555545-file-uploads-with-gpts-and-advanced-data-analysis-in-chatgpt.

[34] OpenAI (2023). Openai Tokenizer. https://platform.openai.com/tokenizer.

[35] Rawte V, Sheth A, Das A (2023). A survey of hallucination in large foundation models. http://arxiv.org/abs/2309.05922.

[36] Ji Z, Lee N, Frieske R (2022). Survey of hallucination in natural language generation. http://arxiv.org/abs/2202.03629.

[37] Shuster K, Poff S, Chen M Retrieval augmentation reduces hallucination in conversation. https://aclanthology.org/2021.findings-emnlp.

[38] Masic I, Miokovic M, Muhamedagic B (2008). Evidence based medicine - new approaches and challenges. Acta Inform Med.

[39] Woolf SH, Grol R, Hutchinson A (1999). Clinical guidelines: potential benefits, limitations, and harms of clinical guidelines. BMJ.

[40] Graham R, Mancher M, Wolman DM (2011). Current best practices and proposed standards for development of trustworthy Cpgs: part 1, getting started.

[41] Prince M (2004). Does active learning work? A review of the research. *J of Engineering Edu*.

[42] The Royal College of Ophthalmologists (2012). Diabetic retinopathy guidelines. https://www.rcophth.ac.uk/resources-listing/diabetic-retinopathy-guidelines/.

[43] (2022). The Royal college of Ophthalmologists. management of angle-closure glaucoma guideline. https://www.rcophth.ac.uk/resources-listing/management-of-angle-closure-glaucoma-guideline/.

[44] Khanani AM, Patel SS, Staurenghi G (2023). Efficacy and safety of Avacincaptad Pegol in patients with geographic atrophy (Gather2): 12-month results from a randomised, double-masked, phase 3 trial. Lancet.

[45] Heier JS, Lad EM, Holz FG (2023). Pegcetacoplan for the treatment of geographic atrophy secondary to age-related macular degeneration (OAKS and DERBY): two Multicentre, randomised, double-masked, sham-controlled, phase 3 trials. Lancet.

[46] Boyer DS, Schmidt-Erfurth U, van Lookeren Campagne M (2017). The pathophysiology of geographic atrophy secondary to age-related macular degeneration and the complement pathway as a therapeutic target. *Retina*.

[47] ASRS Research and Safety in Therapeutics (ReST) Committee (2023). Rest committee update on Intraocular inflammation (IOI). https://www.asrs.org/content/documents/asrs-rest-committee-update-on-intraocular-inflammation-after-ivi-2023.pdf.

[48] Chew EY (2023). Complement inhibitors for the treatment of geographic atrophy. Lancet.

[49] Tan TF, Thirunavukarasu AJ, Campbell JP (2023). Generative artificial intelligence through Chatgpt and other large language models in Ophthalmology: clinical applications and challenges. Ophthalmol Sci.

[50] Anthropic PBC (2023). Anthropic. https://www.anthropic.com/index/claude-2-1.

[51] Tang L, Sun Z, Idnay B (2023). Evaluating large language models on medical evidence summarization. NPJ Digit Med.

[52] Haupt CE, Marks M (2023). AI-generated medical advice-GPT and beyond. JAMA.

[53] Anthropic PBC (2022). Anthropic. https://www.anthropic.com/product.

[54] OpenAI (2023). Introducing Chatgpt enterprise. https://openai.com/blog/introducing-chatgpt-enterprise.

[55] Meskó B, Topol EJ (2023). The imperative for regulatory oversight of large language models (or Generative AI) in Healthcare. *NPJ Digit Med*.

[56] Reddy S (2023). Evaluating large language models for use in Healthcare: A framework for Translational value assessment. Informatics in Medicine Unlocked.

[57] Harrer S (2023). Attention is not all you need: the complicated case of ethically using large language models in Healthcare and medicine. EBioMedicine.

[58] Goddard K, Roudsari A, Wyatt JC (2012). Automation bias: a systematic review of frequency, effect mediators, and Mitigators. J Am Med Inform Assoc.

[59] Gutierrez CT, Loizides C, Hafez I (2023). Acute phase response following pulmonary exposure to soluble and insoluble metal oxide Nanomaterials in mice. *Part Fibre Toxicol*.

[60] Liu NF, Lin K, Hewitt J (2023). Lost in the middle: how language models use long contexts. http://arxiv.org/abs/2307.03172.

[61] Llmtest_Needleinahaystack: doing simple retrieval from LLM models at various context lengths to measure accuracy. https://github.com/gkamradt/LLMTest_NeedleInAHaystack.

[62] Sclar M, Choi Y, Tsvetkov Y Quantifying language models’ sensitivity to spurious features in prompt design or: how I learned to start worrying about prompt Formatting. http://arxiv.org/abs/2310.11324.

